# Ohio College of Dental Surgery

**Published:** 1851-10

**Authors:** 


*Ohio College of Dental Surgery.*—It will be seen by the announcement of the above institution, that Dr. John Allen of Cincinnati, Ohio, has been appointed to the Chair of Operative and Mechanical Dentistry. We congratulate the school on having secured the services of so good a dentist.

